# *Indigofera tinctoria* L. leaf powder promotes initiation of indigo reduction by inducing of rapid transition of the microbial community

**DOI:** 10.3389/fmicb.2022.957809

**Published:** 2022-08-09

**Authors:** Helena de Fátima Silva Lopes, Zhihao Tu, Hisako Sumi, Isao Yumoto

**Affiliations:** ^1^Bioproduction Research Institute, National Institute of Advanced Industrial Science and Technology (AIST), Sapporo, Japan; ^2^Laboratory of Environmental Microbiology, Graduate School of Agriculture, Hokkaido University, Sapporo, Japan; ^3^North-Indigo Textile Arts Studio, Otaru, Japan

**Keywords:** indigo reduction ecosystem, *Indigofera tinctoria* L. leaf powder, *Alkalihalobacillus*, *Polygonibacillus*, alkaliphilic bacteria, *Bacillaceae*, phytochemicals

## Abstract

Water-insoluble indigo is solubilized by the reducing action of microorganisms which occurs during fermentation. In natural indigo fermentation, composted leaves of *Polygonum tinctorium* L. (*sukumo*) are the raw material that has been used as both the indigo source and the bacterial inoculum. Ideally, indigo reduction occurs shortly after preparation of the fermentation vat. The time-to-reduction depends on the quality of the *sukumo* and the methods for preparation and management of the fermentation batch. We estimated the effect of adding *Indigofera tinctoria* L. leaf powder (LP) to indigo fermentation in two fermentations originally exhibiting either rapid or slow time-to-reduction (T-*sukumo* and D-*sukumo*, respectively). *Alkalihalobacillus* spp. (97.7%–98.4% similarities with *Alkalihalobacillus macyae*) were observed only in the LP-added T-*sukumo* fermentation liquor. They appeared from day 1 (0.7%) and increased to 24.4% on day 6, and their presence was related to indigo reduction. Differences in functional ratio between LP-added and its control batches revealed enhancement of pathways related to reconstitution of cellular functions and substrate metabolisms, to all of which *Alkalihalobacillus* spp. contributed intensively. In D-*sukumo* batch, appearance of bacteria necessary to initiate indigo reduction (principally *Anaerobacillus*/*Polygonibacillus*) was comparatively slower. LP promotes earlier indigo reduction in both T- and D-*sukumo*-based batches, owing to its promotion of microbiota transition. The effect of the LP was intensified from day 1 to day 2 in both *sukumo* using batches according to the assumed function of the microbiota. The initial effect of LP on the T-*sukumo* batches was more intense than that in the D-*sukumo* batches and was continued until day 3, while the duration in the T-*sukumo* batches was continued until day 5. Based on these observations, we propose that the LP functions through its phytochemicals that eliminate oxygen, stimulate the microbiota, and accelerate its transitional changes toward a suitable function that opens the pathway for the extracellular electron transfer using carbohydrates as a substrate.

## Introduction

Indigo has been used as a dye material for thousands of years (Splitstoser et al., 2016). It is contained in various plants, including woad (*Isatis tinctoria* L.) which has been used in Europe and southeastern Russia; knotweed [*Persicaria tinctoria* (Ation) Spach] which has been used in China, Korea and Japan; true indigo (*Indigofera tinctoria* L.) and Guatemalan indigo (*Indigofera suffruticosa* Mill.) which have been used in India; and Ryukyu-ai [*Strobilanthes cusia* (Nees) Kuntze] which has been used in Hainan island, China and Okinawa Prefecture, Japan (Cardon, 2007; Chavan, 2015; Zhang et al., 2019; Lopes et al., 2021b). Precipitated indigo, which indigo itself after extraction from plants, has become popular and has been used as source of indigo dye in India, China, Southeast Asia and Okinawa Prefecture, Japan. Extracted dye scarcely contains the source microorganisms that are necessary for the successive step of indigo fermentation for indigo reduction. Therefore, seed microorganisms for example in previous fermentation fluid should be added to perform prompt indigo reduction by fermentation.

In addition to the extraction of indigo, procedures involving composting of plants containing indigo have been performed in Europe and Asia. A composting procedure in Europe dates back to the Middle Ages. Harvested woad leaves are cut into very small pieces and pounded to a moist material by wooden posts in a tub, then gathered into balls of 5–8 cm in size. The prepared balls are fermented for a short period (~4–7 days) under appropriate moisture conditions and dried in the sun. The resulting balls are crushed, and the products are couched over a long period (~40 days) under appropriate moisture for the decomposition of the plant by microbial activity. The temperature of couching woad rises to over 50°C. Therefore, resultant couched woad is subsequently transferred to a woad vat for a fermentation to reduce the indigo (Hartl et al., 2015).

*Sukumo* is a traditional indigo-dyeing material in Japan prepared by composting *Polygonum tinctorium* L (Aino et al., 2018). The *P. tinctorium* L. leaves are piled on an indoor earthen floor (approximately 5 m × 5 m) to a height of ~1 m. Appropriate conditions for the activity of the microorganisms involved fermentation must be maintained for ~100 days. Maintenance of fermentation conditions includes maintaining a high temperature of approximately 70°C, turnover of the piles of the leaves to introduce air, and adjusting moisture content by adding water. This process requires the outstanding technical skills of trained and well-experienced craft workers. During the fermentation process the constituents of the leaves are appropriately digested by microorganisms and indican contained in the leaves is oxidized to indigo.

The coached woad and *sukumo* are not only produced as the source of indigo but also are used as bacterial seeds in the following liquid fermentation (Aino et al., 2018). Furthermore, the remnant plant material, deceased cells of microorganisms, and metabolic byproducts of microorganisms involved in the composting processes may be utilized as the nutrients for microorganisms in the indigo reducing fermentation. In addition to these multiple-functions, coached woad and *sukumo* exhibit exquisite functional preservation for the indigo reducing fermentation. For example, 10 years old *sukumo* can still be utilizable appropriately for successive fermentations.

Since prior initiation of indigo reduction is desirable to maximize the usability of the fermentation fluid, changes in microbiota at the initial fermentation phase have been studied *via* clone library analysis (Aino et al., 2010) and next generation sequencing (Tu et al., 2019a,b, 2021). At the beginning of fermentation, oxygen-metabolizing-bacteria (e.g., facultative anaerobic *Bacillaceae*) first appear. They consume oxygen and their ratio decrease with the decrease in redox potential in the fluid, while the ratios of other bacteria (e.g., other facultative anaerobic *Bacillaceae* and obligate anaerobic *Proteinivoraceae*) increase. Within 5 days after the preparation, the ratio of facultative anaerobes decrease, and dominance of obligate anaerobes increases, and *Amphibacillus* spp. and/or *Alkalibacterium* spp. appear at ~day 4–8. At this state, indigo reduction is initiated. However, the mechanisms and requirements for initiation of indigo reduction have not been clarified to our knowledge.

Although it is difficult to regulate microbiota to a desirable state, the microbiota in indigo fermentation can be regulated in a manner suitable for indigo reduction, acceleration of initiation of indigo reduction will be possible (Lopes et al., 2021a). We found that addition of *Indigofera tinctoria* L. leaf powder (LP) to the fluid at the beginning of its fermentation reduced redox potential and promoted the appropriate transition in the microbiota of the fluid.

In this study, we aim to understand the reason for the LP reducing the redox potential by estimating the oxygen consumption ability of the LP. We try to understand the required state of the microbiota and the associated required metabolic basis for indigo reduction by producing differentiated the period form the fermentation preparation to the initiation of indigo reduction, by using different kinds of *sukumo* combined with additives (LP and wheat bran), and analyzing the microbiota and estimating the predictive function of the metagenomes. The results offer procedures to regulate the microbiota to desirable states and manage the bacteria function to initiate indigo reduction.

## Materials and methods

### Preparation of *sukumo* fermentation fluid for rapid-changing microbiota

A small-scale batch fermentation (l L) for rapid-changing microbiota was prepared by using *sukumo* produced in Tokushima, Shikoku, Japan by A.S. (T-*sukumo*). Indigo reduction was initiated on day 5 (normally day 4 in ideal preparation procedure) from the preparation fermented using this *sukumo*. The *sukumo* fermentation fluid was prepared using *sukumo* and an alkaline solution made of wood ash extract. The wood ash extract was produced by mixing of 380 g wood ash (Quercus phillyraeoides A. Gray, Nagomi Co., Gobo, Wakayama, Japan) and 5 L tap water, and boiling for 10 min. The supernatant was carefully decanted into 1 l Erlenmeyer flasks. After the wood ash extract cooled to 60°C, 76 g *sukumo* was added to it. When the fluid cooled to 30°C, *Indigofera tinctoria* L. leaf powder (LP; NCC Agro Industries, Tamil Nadu, India; 0.5 g) which was for hair dye was added. LP is manufactured by air-drying leaves and grinding them into fine powder. This batch was labeled LP-added. The flask containing no LP was the control. Batches T1 and T2 were control (no additive) and LP-added batches, respectively. Replicate batches were prepared to confirm the effect of LP on T-*sukumo*. Sample preparation, fermentation period, samples for microbiota analysis and the pH maintenance are summarized in Table 1.

**Table 1:** Preparation and maintenance procedures for six batches of *sukumo* fermentation fluids.

Batch	*Sukumo*	Leaf powder (LP)	Wheat bran	Observed fermentation period	Samples for analysis of microbiota	pH range during fermentation
T1	T-*sukumo*	−	−	Days 1–27	Days 1–6 (6)^*^	9.9–10.3
T2	T-*sukumo*	+	−	Days 1–27	Days 1–6 (6)	9.8–10.2
D1	D-*sukumo*	−	−	Days 1–32	Days 1, 2, 3,5 7, 11, 21 (7)	10.3–10.7
D2	D-*sukumo*	+	−	Days 1–32	Days 1, 2, 3,5 7, 11, 21 (7)	10.2–10.8
D3	D-*sukumo*	−	+	Days 1–32	Days 1, 2, 3, 4, 5 6, 11, 21 (8)	10.2–10.7
D4	D-*sukumo*	+	+	Days 1–32	Days 1, 2, 3, 4, 5 6, 11, 21 (8)	10.1–10.6

* Sample number.

### Preparation of *sukumo* fermentation fluid for slow-changing microbiota

A small-scale fermentation batch (l L) for slow-changing microbiota was prepared using *sukumo* manufactured by K.S. (D-*sukumo*; Date, Hokkaido, Japan). Indigo reduction was initiated later than day 10 from fermentation fluid prepared using this *sukumo*. In a previous report, it took 58 days for obvious dyeing to appear (Lopes et al., 2021a). The *sukumo* mixed with wood ash extract as described above produced four different batches. Batches D1 and D2 were control (no additives) and 0.5 g of LP added, respectively. In batches D3 and D4, wheat bran was added at day 4 and LP wad also added to batch D4 (when its preparation was ready).

### Maintenance and measurement of prepared *sukumo* fermentation batches

The effects of LP on slow-changing microbiota indigo fermentation employed *sukumo* produced in Date, Hokkaido, Japan (D-*sukumo*) have already been studied (Lopes et al., 2021a). However, the pH of the fermentation fluid fluctuated between pH 10.5 and pH 11.8. We used Ca(OH)_2_ for adjustment in that study because it is often used in the fluid for indigo dyeing. Uncertainty about the pH adjustment by Ca(OH)_2_ is attributed to its immediate insolubility in the fluid. Therefore, it is difficult to know accurately what amount is required for the pH adjustment. In the present study, we used NaOH for adjustment of the pH of fermentation to achieve an even more accurate regulation of pH. The fermentation fluid pH was maintained between 10.0 and 10.7 with NaOH. The flasks were kept at 26°C in a temperature regulated room and gently stirred once a day. The pH and redox potential (ORP) of the fermentation fluids were measured with a D-71 pH meter (Horiba, Kyoto, Japan) and a D-75 pH/ORP/DO meter (Horiba), respectively. To check indigo reduction, a piece of small cotton cloth (approximately 3 cm × 4 cm) was dipped in the fermentation fluid for 1 min and then, exposed to air for 10 min to oxidize the indigo dye, then stored in the dark. Replicate batch was not prepared, since each experimental condition contained overlapping with the others and the effect of LP on D-*sukumo* had been confirmed in other experiments. Sample preparation, fermentation period, samples for microbiota analysis, and pH maintenance are summarized in Table 1.

### DNA extraction and PCR

Each *sukumo* fermentation fluid, with or without additives such as LP or wheat bran, was centrifuged at 15,000×*g* for 10 min at 25°C to obtain a pellet to be used for DNA extraction. The obtained pellet was treated with ISOIL extraction kit (Nippon Gene, Tokyo, Japan) According to the manufacturer’s instruction except for the final step, when 50 μl Tris-EDTA (TE) was added instead of 100 μl. Samples from two *sukumo* (T-*sukumo* and D-*sukumo*) batches were directly treated by the kit. The extracted DNA was stored at −35°C until use. The bacterial 16S rRNA gene sequence of the V3–V4 region (341F–805R) was amplified with primer pair: V3V4f_MIX (ACACTCTTTCCCTACACGACGACGCTCTTCC-GATCT-NNNN-CCTACGGG-NGGCWGCAG) and V3V4r_MIX (GTGACTGGAGTTCAGACGTG-TGCTCTTCCGATCT-NNNNN-GACTACHVGGGTATCTAATCC) Purchased from Bioengineering Lab. Co. Ltd. (Sagamihara, Kanagawa, Japan). The primers consisted of an adapter sequence followed by insertions with 0–5 bases of random sequences (described as N) (adaptor) and a sequence for amplification of the targeted 16S rRNA sequence. The random sequences were set to improve quality. the PCR solution (40 μl) consisted of 4 μl of 10× Ex buffer (Takara Bio, Otsu, Shiga, Japan), 3.2 μl dNTPs (Takara Bio, Otsu, Japan), 2 μl forward primer, 2 μl reverse primer, 2 ng DNA template (extracted sample), 0.4 μl of 5 U·ml^−1^ Ex Taq polymerase (Takara Bio). The amplification reaction of the targeted sequence was performed as follows: 94°C for 2 min; 25 cycles of 94°C for 30 s, 55°C for 30 s and 72°C for 30 s; extension of 72°C for 5 min. Product identity and quality were confirmed by agarose gel Electrophoresis.

### Next generation sequencing

The second PCR was performed by Bioengineering Lab. Co. Ltd. The first PCR product was amplified with a tailed primer set and the product was purified. The next generation sequencing (NSG) was conducted on an Illumina MiSeq platform (Illumina, San Diego, CA United States). The raw reads were pre-processed by Bioengineering Lab. Co. Ltd. and delivered as fastq.gz output files. The gene sequences of primers and adaptors in the fastq.gz files were removed by Cutadapt version 1.18 and processed by QIIME2 ver. 2020.2 (Bolyen et al., 2019). Paired end read merge, construction of operational taxonomic unit (OTU) and quality control were performed by using the Divisive Amplicon Denoising Algorithm (DADA2; Callahan, 2016). Taxonomic classification was achieved using the feature classifier for 16S rRNA based on the primer pair 341F–805R prepared based on the SILVA database (Quast et al., 2013; Yilmaz et al., 2014). For more updated taxonomic identification, the representative sequences were applied to a BLAST search in NCBI (Miura et al., 2019). Rarefaction curves of observed OTUs and the Shannon index of diversity was plotted according to QIIME2 alpha diversity script. The results of the Bray–Curtis principal coordinate analysis (PCoA) were plotted using MicrobiomeAnalyst (Chong et al., 2020). Predictive functions of the metagenomes were estimated using PICRUSt2 (Douglas et al., 2020). Based on the PICRUSt2 results, taxa-functional relationships were analyzed by BURRITO (McNally et al., 2018).

### Oxygen consumption by leaf powder

Wood ash extract was prepared as described above. Samples of 2 g of leaf powder were dissolved in 40 ml of wood ash extract (pH 11.5) in 68 ml-volume vials with rubber stoppers (Nichiden-rika glass, Kobe, Japan). All bottles were sealed right after the addition of leaf powder and incubated with agitation of 180 rpm at 27°C. The gas phase was measured hourly using a gas chromatograph (GC-2014; Shimadzu, Kyoto, Japan) equipped with a thermal conductivity detector and molecular sieve 13X column (Shimadzu; 2.0 m × 3.0 mm ID). The column, injection, and detector temperatures were 70°C, 180°C, and 200°C, respectively (Kato et al., 2014). For the negative control, wood ash extract was used. The treatment and the control were made in triplicate analysis.

## Results

### Effect of *Indigofera tinctoria* L. leaf powder on ORP and the microbiota in indigo fermentation fluid

LP acts to accelerate indigo reduction in the natural fermentation of D-*sukumo*, which produces slow change in microbiota during fermentation (Lopes et al., 2021a). In this study, we aim to confirm the accelerative effect of LP on microbiota using T-*sukumo*, which without LP would produce rapid change in microbiota, and D-*sukumo*, which without LP would produce slow change in microbiota.

To estimate the capacity of LP to eliminate oxygen in the fermentation fluid directly, a large amount of LP was added to wood ash extract and changes in oxygen concentration in the fluid were estimated (Supplementary Figure S1). The oxygen in the LP-added fluid disappeared in 7 h while that in the no-LP-added batch scarcely changed. Thus, the oxygen scavenging effect of LP was demonstrated. LP contains indican, which is the precursor of indigo. Indican in LP is rapidly converted to indigo when in contact with dissolved oxygen. Therefore, indican in LP can contribute to the consumption of dissolved oxygen in the fluid.

Raw sequences (519,743) were obtained from a total of 12 samples, one from each day (day 1 to day 6) after fermentation preparation, from the LP-added and control batches using T-*sukumo*. After adapter trimming, filtering for quality checking, and chimeric exclusion, 355,094 sequences were obtained. Raw sequences (1,579,972) were obtained from a total of 30 samples from the LP-added and control each day from day 1 to day 21 after fermentation preparation from D-*sukumo* used batches. After adapter trimming, filtering for quality checking, and chimeric exclusion, 870,304 sequences were obtained.

**Figure 1:**
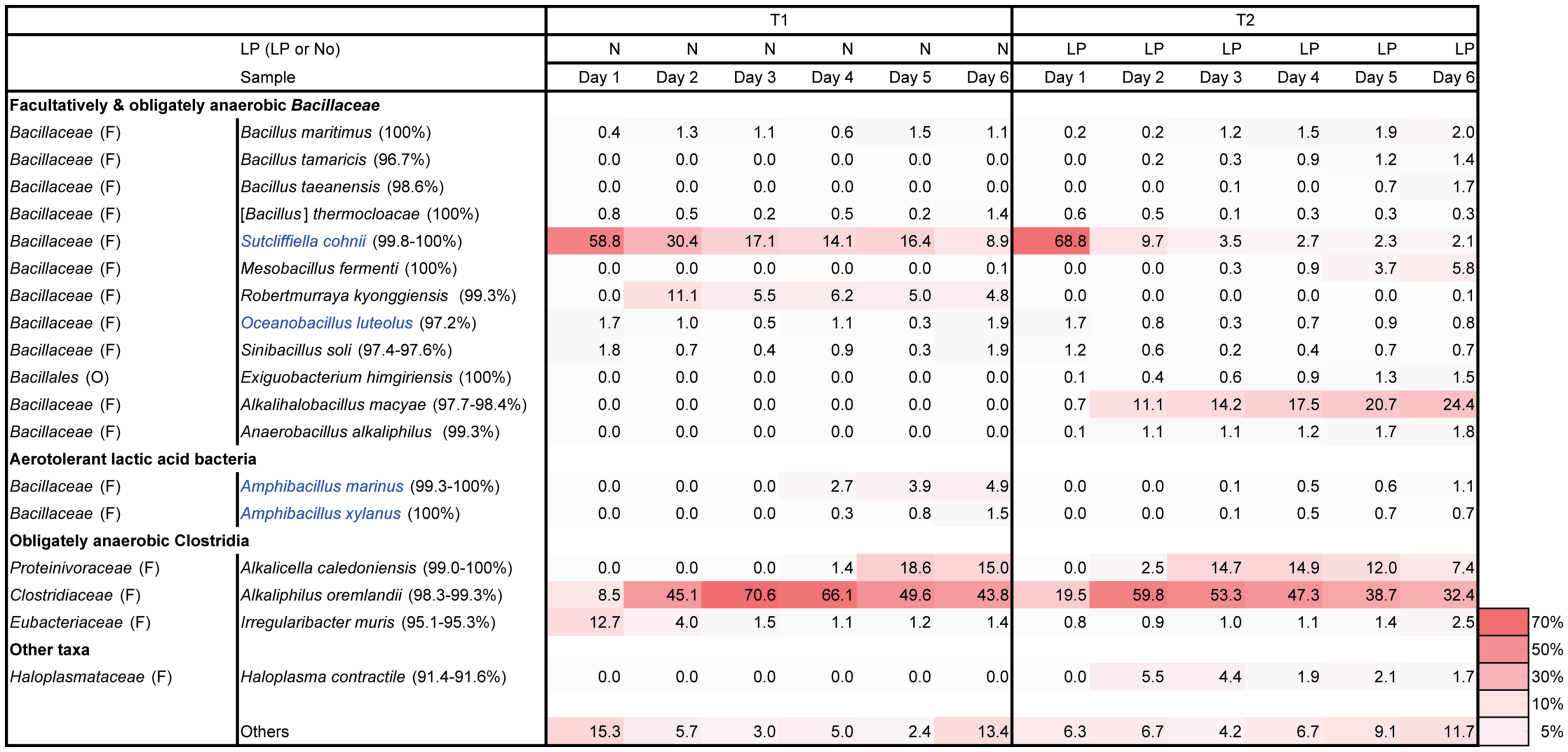
Changes in the relative abundance of bacterial communities (≥1.4% in any sample), based on 16S rRNA analysis. Blue letters indicate the confirmed indigo-reducing taxa (including unpublished results). The percentages in the brackets indicate the similarities with the known species in the NCBI database.

**Figure 2:**
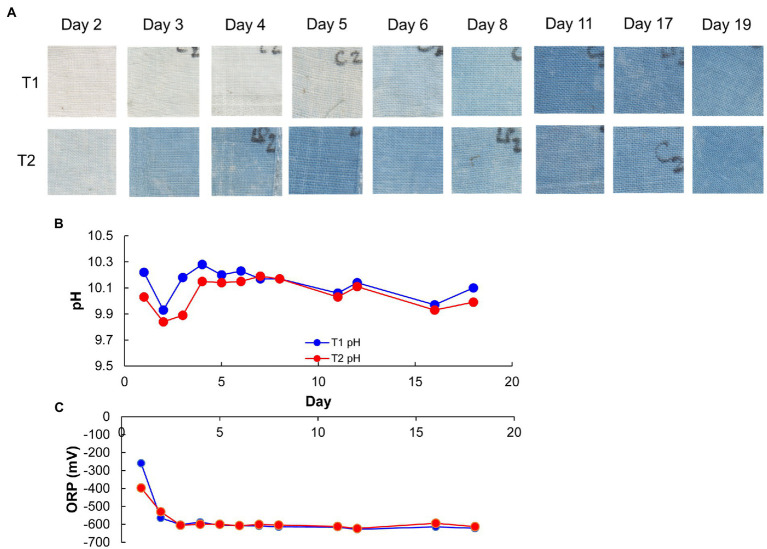
Dyeing results **(A)**, pH **(B)**, and redox potential **(C)**, depending on the fermentation period in indigo fermentation fluid batch T2 (*Indigofera tinctoria* L. leaf powder [LP] added) T1 (control) using T-*sukumo*. Blue and red circles indicate T1 and T2, respectively, in **(B,C)**.

Changes in pH, ORP, dyeing intensity, and bacterial community in the fermentation using T-*sukumo* are shown in Figures 1, 2. The pH was maintained between 9.8 and10.3. The reduction in ORP was faster in the LP-added batch and reduction in ORP may have the effect of converging the diversity of the microbial flora, also reflected in the lower percentage of “others” in the T2 batch at day 1. Although faint dyeing was observed at day 2 in the LP-added batch (T2), an evident difference in dyeing between the T2 batch and its control (T1) was observed at day 3. Facultative anaerobic alkaliphile *Sutcliffiella cohnii* predominantly appeared on day 1 in both the LP-added batch and the control. The speed of its disappearance was greater in the LP-added batch. *S. cohnii* decreased on day 2, and obligate anaerobic *Alkaliphilus* spp. appeared in both batches. The changes may be attributed to the decreasing ORP due to the aerobic metabolism of the initially colonizing microbiota. While obligate anaerobic *Alkalihalobacillus* spp. (*Alkalihalobacillus macyae* [97.7%–98.4% similarity]) appeared (11.1%) in the T2 batch, facultative anaerobic *Robertmurraya kyonggiensis* (99.3% similarity; 11.1%) appeared in the control. In addition, the obligate anaerobic *Alkalicella caledoniensis* (99.0%–100%) appeared 2 days earlier in the T2 than in the T1 batch. Although the deference in ORP between T2 and T1 batches appears small, it indicates that the environment in the T2 batch was more favorable for obligate anaerobes. The pronounced difference in the microbial community between the T2 and T1 batches was the appearance of *Alkalihalobacillus* spp. in the LP-added batch. It seems that the *Alkalihalobacillus* spp. ratio contributed to the dyeing intensity. On the other hand, increasing the ratio of *Amphibacillus* spp., which has been reported to be indigo reducers may have contributed to dyeing intensity in the T1 batch after day 5.

**Figure 3:**
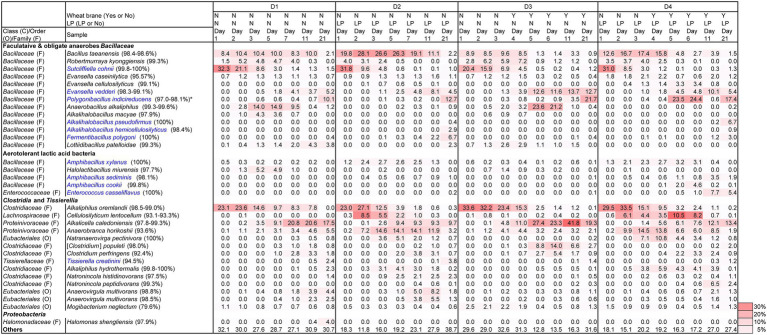
Changes in the relative abundance of bacterial communities (≥2.0% in any sample) depending on the fermentation period in indigo fermentation fluid batches D2 and D4 (*Indigofera tinctoria* L. leaf powder [LP] added) and D1 and D3 (controls) and using D-*sukumo*, based on 16S rRNA analysis. Wheat bran was added to batches D3 and D4 at day 4. Blue letters indicate the confirmed indigo-reducing taxa (including unpublished results). The percentages in the brackets indicate the similarities with the known species in the NCBI database. ^*^Anaerobacillus alkaliphilus-like taxa are involve in *Polygonibacillus indicireducens* in batch D4.

**Figure 4:**
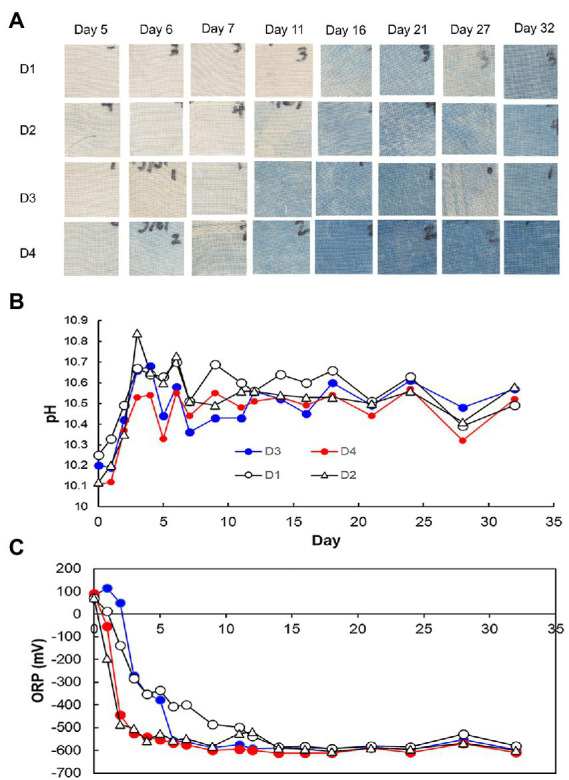
Dyeing results **(A)**, pH **(B)**, and redox potential **(C)**, depending on the fermentation period in indigo fermentation fluid using D-*sukumo* with the addition of *Indigofera tinctoria* L. leaf powder (LP) added [D2: open triangles and D4: red circles in **(B,C)**] and controls [D1: open circles and D3: blue circles in **(B,C)**]. Wheat bran was added to batches D3 and D4 on day 4.

Changes in pH, ORP, dyeing intensity, and bacterial community, in the fermentation using D-*sukumo* are shown in Figures 3, 4. The pH range kept between pH 10.1 and pH 10.8. A rapid decrease in ORP was observed in the D2 batch (first decrease in ORP *ca.* −503 mV at day 3), while it took 11 days to decrease the ORP *ca.* −499 mV in the D1 batch. However, although the cloth was faintly dyed on day 5 in D2 batch, the intensity decreased up to day 7 and faintly dyeing was observed at day 11 again. This is probably explained by the exhaustion of the readily utilizable substrates that are utilized by indigo-reducing bacteria. The obvious dyeing was observed in both D2 and D1 batches at day 21. This may be attributed to the appearance of *Polygonibacillus indicireducens* (97.0%–98.1%), which is supposed to be a bacterium that can use hard-to-utilize substrates. Higher ratio of the reported indigo-reducing *Fermentibacillus polygoni* was observed in the LP-added batch D2. We propose that the microbial community and its succession occurred in the D2 batch when readily utilizable substrates were exhausted for the corresponding microbial community.

Differences between wheat the bran-added batch (D3) and the LP and wheat bran-added batch (D4) are attributable to the addition of LP in the presence of wheat bran after day 4. Therefore, we expect the effect of wheat bran to appear after day 5. The pH range was maintained between 10.1 and10.6. In this case also, we observed faster decrease in ORP in the LP-added D4 batch than in the D3 batch (control). By adding both LP and wheat bran, the ratio of aerotolerant lactic acid bacteria such as *Amphibacillus* spp. and *Enterococcus casseliflavus* was increased. The taxa were identified as indigo reducers. However, the environmental factors for propagating these taxa should be clarified in future studies. The latter taxon was rarely observed in the previous studies using D-*sukumo* only (Lopes et al., 2021a). The reported indigo-reducing *Alkalihalobacillus pseudofirmus* was observed only in the LP-added batch D4. Although dyeing intensities in batches D1, D2, and D3 fluctuated within 32 days, the batch D4 maintained strong dyeing even after day 16 (Figure 4). In summary, the results can be demonstrated that addition of both LP accelerated the initiation of indigo reduction and addition of wheat bran stabilized the dyeing intensity by suppling substrates to the indigo reducing bacteria.

### Effect of *Indigofera tinctoria* L. leaf powder on changes in alpha and beta diversities

Changes in alpha diversity at a sampling depth of 10,886 from the rarefaction curves based on the observed OTUs in batches T1 and T2 are shown in Supplementary Figure S2. The lesser-observed OTUs in the T2 batch compared to the T1 batch suggested the suppression of microorganisms requiring oxygen for their metabolisms or microorganisms sensitive to phytochemicals in the LP. Although a drastic change occurred in the microbial community from day 1 to day 2 in the T2 batch (Figure 1), the observed OTUs were slightly decreased, while the Shannon index was increased. On the other hand, the observed OTUs of the T1 batch tended to decrease from day 1 to day 3 and from day 4 to day 5. The Shannon index exhibited a decreased only from day 2 to day 3. The deceasing may be related to the consumption of the readily utilizable substrates derived from *sukumo*. The exhaustion of substrates may be attributed to continue dominance of *S. cohnii* (99.8%–100%) and the appearance of *Robertmurraya kyonggiensis* (99.3%) in the T1 batch. A dramatic increase was observed in the OTUs from day 5 to day 6 in the T1 batch. This corresponded to the increase in *Amphibacillus* spp. and others. This increase may be attributed to changing mainly used substrates by the bacteria in the microbiota due to the exhaustion of readily utilizable substrates, and as a consequence the initiation of indigo reduction was initiated.

**Figure 5:**
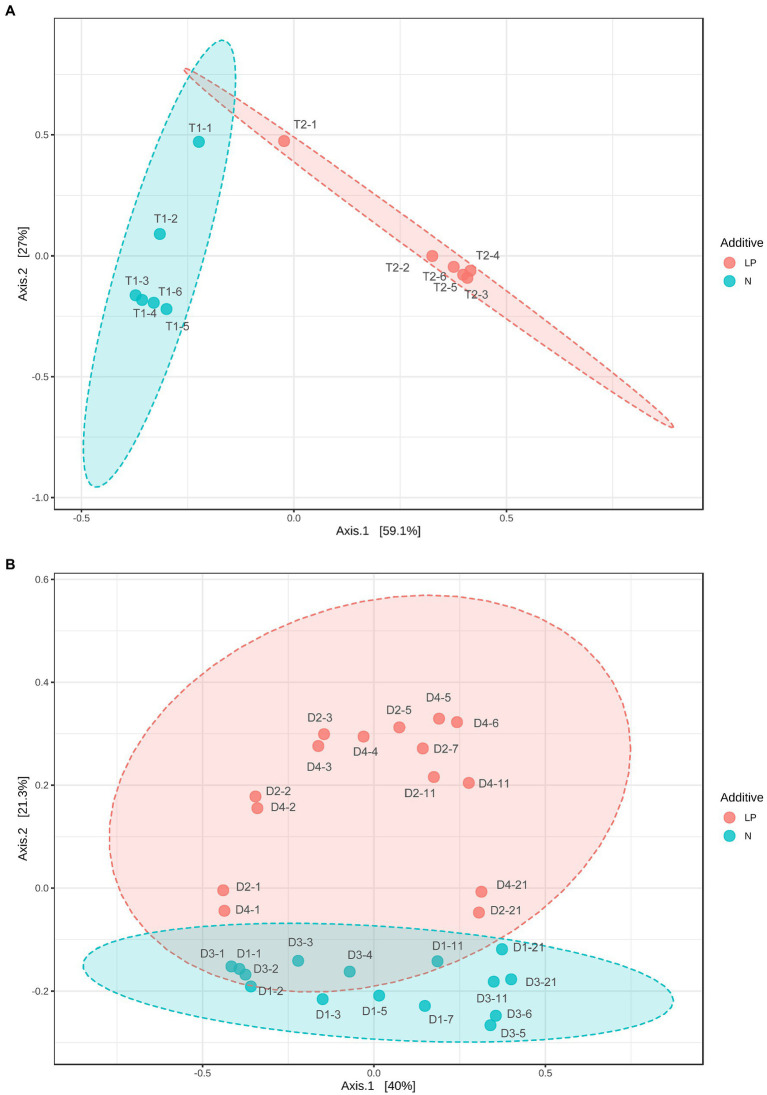
Bray–Curtis principal coordinate analysis (PCoA) of indigo fermentation using T-*sukumo* **(A)** and D-*sukumo* **(B)**. Branch numbers indicate the number of days of fermentation. **(A)** *Indigofera tinctoria* L. leaf powder (LP) added (T2: red circles) and control (T1: blue circles). **(B)** *s* L. leaf powder (LP) added (D2 and D4: red circles) and controls (D1 and D3: blue circles). Wheat bran was added to batches D3 and D4 on day 4.

The relative difference in changes in microbiota between the T2 and T1 batches was estimated using Bray–Curtis PCoA (Figure 5). The direction of change of microbiota differed between the T2 and T1 batches. The initial change (from day 1 to day 2) was faster in the T2 than in the T1 batches. The initial change in each batch corresponded to a change in dominance from facultative to obligate anaerobes. Although dyeing intensity increased, and a large increase in observed OTUs were observed from day 5 to day 6 in the T1 batch, they were not reflected in the PCoA. Therefore, observation of multi-faceted factors must be required to account for the observed changes in dyeing intensity.

Changes in alpha diversity at a sampling depth of 12,851 from the rarefaction curve based on the observed OTUs and Shannon index are shown in Supplementary Figure S3. The initial microbial diversity was much higher in the D1 than in the D2 batches and we presumed this attribute to the more rapid decrease in ORP in the D2 batch. Tendency of the observed OTUs to slowly decrease from day 1 to day 7 in the batch D1 is in accordance with the slow decrease in ORP. However, a slight increasing tendency was observed in Shannon index during this period. On the other hand, the microbial diversity increased from day 2 to day 7 in the batch D2. This may be attributed to the microbiota present at day 2 trained to utilize available substrates including deceased cells under the anaerobic alkaline conditions by transition of the microbiota. Moreover, the increasing velocity in the microbial diversity until day 7 tended to decrease after 11. This may be attributed to the paucity of the substrates which initially existed for the corresponding microbial community. Initial change in the trend of the alpha diversities from days 1 to day 3 in the batch D3 was similar to that of the batch D1. We consider the decrease in the observed OTUs and the Shannon index at day 5 in both the batches D3 and D4 to be the effect of the addition of wheat bran. This timing was accordance with the dramatic microbial community change (Figure 3). The observed OTUs tended to increase in the observation period following the impact of the introduction of wheat bran as the microbial community trained to the wheat brane as the substrate.

The relative differences in the changes in microbiota between the LP-added batches (D2 and D4) and the controls (D2 and D3) were estimated using Bray–Curtis PCoA (Figure 5). The initial changes in the microbiota in this slow-changing microbiota fermentation fluid were slower than the rapid-changing microbiota fermentation fluid. As observed for the T-*sukumo*, the LP-added batches (D2 and D4) the direction of change differed from that in the non-LP-added batches and the velocity of change accelerated from day 1 to day 2. The latter effect was reflected in the faster increase in obligate anaerobes in the LP-added batches, which we presumed attribute to the rapid decrease in ORP. Acceleration of microbial community change was also observed in the wheat bran-added batches. The changes in velocity from the D3 day 4 (D3-4) to the D3 day 5 (D3-5) were faster than that of the change in non-wheat bran-added batch change from D1 day 3 (D1-3) to D1 day 5 (D1-5). From the above results, in order to reduce the indigo faster, it is important to make microbiota transitions quickly to the microbial flora that indigo reduction occurs, and the addition of LP and wheat bran is effective for that purpose.

### Effect of *Indigofera tinctoria* L. leaf powder on the induction of various microbial functions

**Figure 6:**
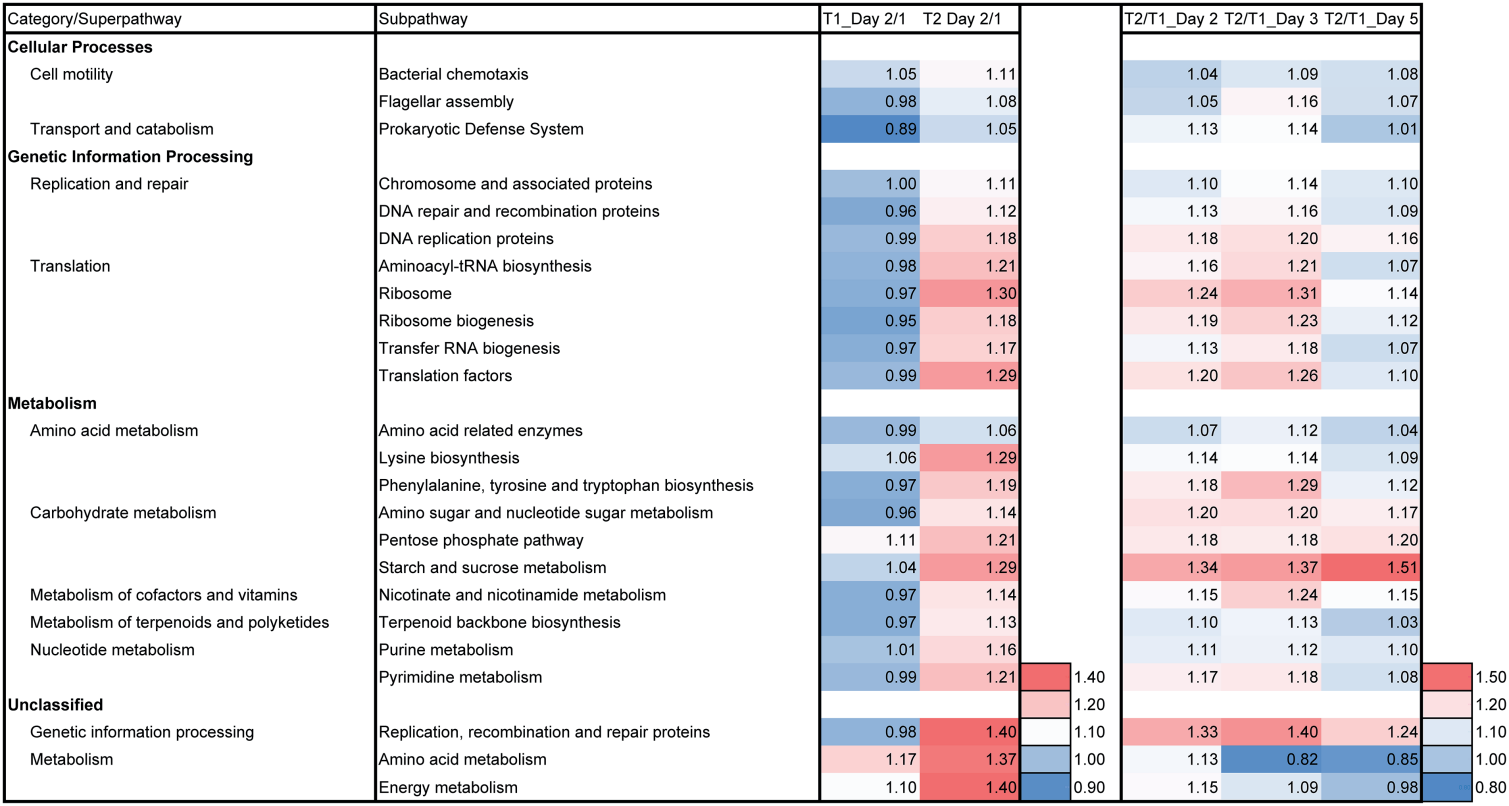
Effects of *Indigofera tinctoria* L. leaf powder (LP) on the ratios (day 2/day 1 and T2/T1 for days 2, 3, and 5) for each relative abundance of functional subpathway of the bacterial communities in the indigo fermentation using T-*sukumo*. The batches of T2 (*I. tinctoria* L. leaf powder [LP] added) and T1 (control) using T-*sukumo* are shown. In addition, the metagenomic prediction produced using PICRUSt2 and BURRITO is shown. Subpathways containing ratios higher than 1.10 in either day 2/day 1 or T2/T1 (for days 2, 5, and 5) were selected.

Predictive functions of the metagenomes were estimated to understand the effect of LP on microbial functions. The day 2/day 1 ratio of batches T1 and T2; and batch LP-added/control (T2/T1) ratio for days 2, 3, and 5 based on the relative functional abundancies for each subpathway revealed the differences between batches T2 and T1 in the predicted metabolic functions and these differences are shown in Figure 6. The intensity of the metabolic change from day 1 to day 2 was relatively large in the T2 batch compared with that in the T1 batch. The enhancement of the superpathways related to the reconstitution of the cellular function and substrate metabolisms more pronounced in batch T2 than that in batch T1. The effect of LP for initial change (day 2/day 1) and in T2/T1 in days 2–5 (Figure 6) as follows: cell motility (two subpathways; e.g., flagella assembly), transport and catabolism (one subpathway; e.g., prokaryotic defense system), replication and repair (three subpathways; e.g., DNA repair and recombination proteins), translation (five subpathways; e.g., ribosome and ribosome biogenesis), amino acid metabolism (three subpathways; e.g., phenylalanine, tyrosine and tryptophan biosynthesis), carbohydrate metabolism (three subpathways; e.g., pentose phosphate pathway), metabolism of cofactors and vitamins (one subpathway; e.g., nicotinate and nicotinamide metabolism), metabolism of terpenoids and polyketides (one subpathway; e.g., terpenoid backbone biosynthesis), nucleotide metabolisms (two subpathways; e.g., purine metabolism), (unclassified) genetic information processing (one subpathways; e.g., replication, recombination and repair proteins), and (unclassified) metabolism (two subpathways; e.g., energy metabolism). It can be noticed that most of the stimulated functional subpathway continued until day 3. For instance, “amino acid metabolism” and “energy metabolism” were strongly enhanced on day 2 in batch T2, while these subpathways were not enhanced on day 5. This result suggested that the duration of the initial effects owing to the LP addition on the T-*sukumo* batch is up to the third day. On the other hand, the three subpathways in the carbohydrate metabolism superpathway and the “replication, recombination and repair proteins” subpathway were continuously enhanced in the LP-added batch. However, these enhancements may be produced by secondly effect of produced by LP. This metabolic trend may lead to the transfer of electrons produced by the metabolisms toward the extracellular indigo particles continuously.

Taxonomic constituents receiving higher impact from LP at day 3 were estimated in the metabolic subpathway, exhibiting pronounced changes related to the initiation of indigo reduction (Supplementary Table S1). Utilization of substrates containing proteins was enhanced initially according to the changes in the subpathway of peptidase. However, this tendency shifted to utilization of carbohydrates from day 2 in the LP-added batch. Although the relative abundances of the OTUs of obligate anaerobes, genera *Alkalicella* and *Alkaliphilus*, were high (14.7% and 53.3%, respectively; Figure 1), the contributions of each most predominant OTU for starch and sucrose metabolism were not high (6.8% and 7.5%, respectively). Surprisingly, the most predominant OTU belonging to genus *Alkalihalobacillus* exhibited large contributions in each representative subpathway (34.8%–54.3%). This result is consistent with the superiority of dye intensity in the LP-added batch, which only contained genus *Alkalihalobacillus*.

**Figure 7:**
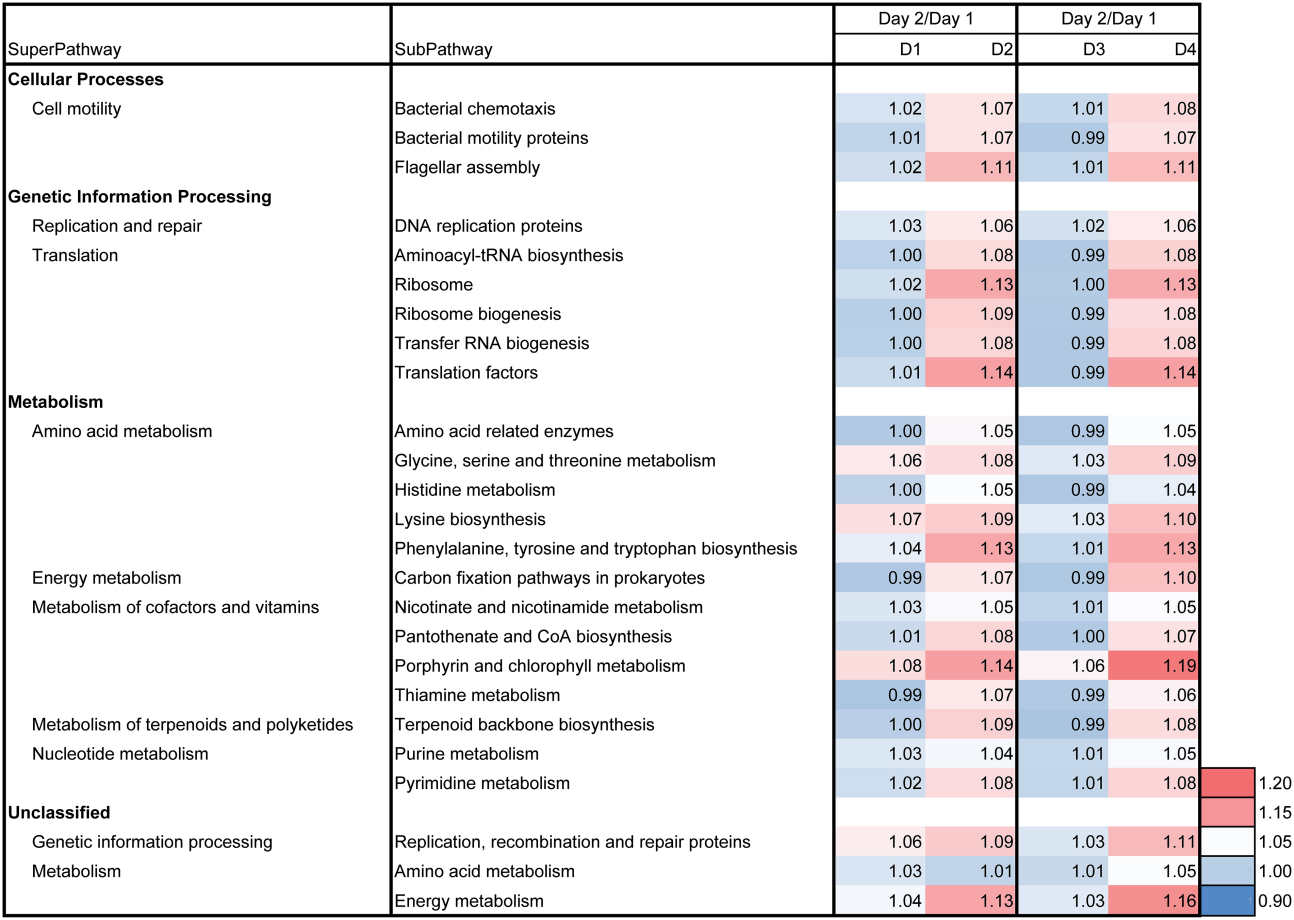
Effects of *Indigofera tinctoria* L. leaf powder (LP) on the ratio (day 2/day 1) for each relative abundance of functional subpathway of the bacterial communities in indigo fermentation using D-*sukumo*. The batches of *I. tinctoria* L. leaf powder (LP) added (D2 and D4) and controls (D1 and D3) are shown. In addition, the metagenomic prediction produced using PICRUSt2 and BURRITO is shown. Subpathways containing ratios higher than 1.05 were selected.

**Figure 8:**
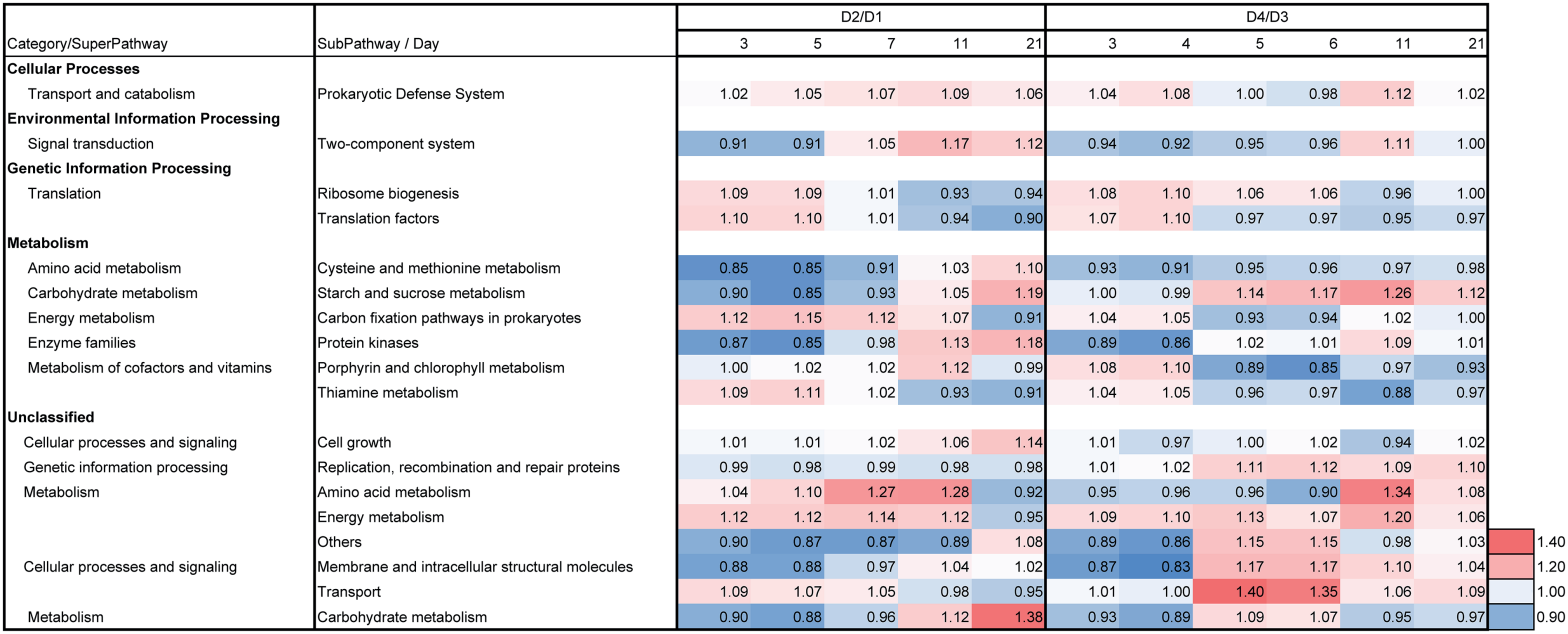
Effects of *Indigofera tinctoria* L. leaf powder (LP) on the ratios (D2/D1 of days 3, 5, 7, 11, and 21; and D4/D3 of days 3, 4, 5, 6, 11, and 21) for each relative abundance of functional subpathway of the bacterial communities in the indigo fermentation using D-*sukumo*. The batches of *I. tinctoria* L. leaf powder (LP) added (D2 and D4) and controls (D1 and D3) are shown. Wheat bran was added to batches D3 and D4 on day 4. In addition, the metagenomic prediction produced using PICRUSt2 and BURRITO is shown. Subpathways containing ratios higher than 1.10 were selected in days 3–21.

The day 2/day 1 ratio and LP-added/control (D2/D1) ratio for day 3, 5, 7, 11, and 21 on the relative functional abundances for each subpathway revealed the differences between batches D2 and D1 in the predicted metabolic functions, as shown in Figures 7, 8. The metabolic changes from day 1 to day 2 was stronger in the batch D2 than the batch D1 as shown in Figure 7. This result is in accordance with the changes in the fermentation batches using T-*sukumo* (Figure 6), and the changes in the microbiota observed in Bray–Curtis PCoA (Figure 5A). The stimulated subpathways by the LP addition from day 1 to day 2, exhibited 72% similarity to that of the fermentation batches using the T-*sukumo*. This finding suggested that LP initially stimulates similar metabolic functions in the microbial community even with a different microbiota. Certain enhanced subpathways were not continuously stimulated after day 5, while “carbon fixation pathways in prokaryotes,” “amino acid metabolism,” and “energy metabolism” were continuously stimulated until day 11 by the LP addition (Figure 8) suggesting that the duration of the initial effects of LP addition is continued until day 5, and the subsequent changes were due to the secondary effects of LP addition. This finding may be related to the increase in the alpha diversity after day 5 (Supplementary Figure S3B). The enhancement of “starch and sucrose metabolism” due to LP addition was observed form day 11, which could be the reason behind the delayed initiation of dyeing in batch D2 compared to batch T2 with T-*sukumo*.

The day 2/day 1 ratio and LP-added/control (D4/D3) ratio for day 3, 5, 7, 11, and 21 on the relative functional abundance for each subpathway revealed differences between D4 and D3 in the predicted metabolic functions, as shown in Figures 7, 8. The stimulated functional subpathways were similar to those of batches D2 and D1 until day 4. Although the enhancements of several functional abundances by LP were transient, the enhancement of “prokaryotic defense system,” “translation factors,” “carbon fixation pathways in prokaryotes” and “porphyrin and chlorophyll metabolism” continued until day 4. This finding suggested that although the duration of the initial effects of LP addition is continued until day 4, its effects were enhanced by wheat bran (introduced on day 4) on day 5. The enhancement of “starch and sucrose metabolism,” “replication, recombination and repair proteins,” “energy metabolism” and “transport” subpathways continued from day 5 until day 21 in the D4 batch suggesting that these functional abundances may be the reason for the sustaining strong indigo reduction in the later phase of indigo fermentation in batch D4 (Figure 4A).

These findings suggest that the high ratio in substrates metabolisms utilizing for sustaining extracellular electron transfer to the total metabolisms of existing microorganisms is important to sustain indigo reduction. Although it is considered that addition of external bacterial substrates may increase microbial species that are unfavorable for indigo reduction, addition of wheat bran induced a favorable change in microbiota; it took ~5 days for induction of apparent indigo reduction according to the observation in the D3 and D4 batches.

Taxonomic constituents exhibiting high contribution to the early metabolic change related to the enhancement by the addition of LP in the D4 batch, were estimated at days 5 (Supplementary Table S2). In all the metabolic subpathways, the contribution of OTUs belonging to *Polygonibacillus*/*Anaerobacillus* was the highest followed by *Evansella* on day 5 except in “energy metabolism” subpathway. Contribution of obligate anaerobes *Cellulosilyticum* and *Natraneroviga*, to “starch and sucrose metabolism” was comparable to the ratio that existed at day 5 (Supplementary Table S2; Figure 3). Given the intensive dyeing observed on day 21 in batch D4, we elucidated the enhanced functional subpathways (Supplementary Figure S4). It was considered that the fermentation phase on day 21 in batch D4 was stable state in the fermentation phase and enhancement subpathways concerned with carbohydrate metabolisms contributed to the dyeing intensity. Taxonomic constituents exhibiting a high contribution for the enhanced functional subpathways on day 21 were estimated Supplementary Table S3. The contribution of OTUs belonging to *Polygonibacillus*/*Anaerobacillus* for the enhanced subpathways was higher than on day 5. The results suggest that the functions involved in contributing indigo reduction are monopolized by specific microbial groups. *Enterococcus* that specifically appeared in the D4 batch that contained both LP and wheat bran contributed subpathways of “ABC transporters,” “transcription factors,” “pentose phosphate pathway,” “starch and sucrose metabolism,” and “function unknown.” Considering the ratio that existed, the contribution of *Enterococcus* to “starch and sucrose metabolism” was high.

### Bacterial community analysis of *sukumo*

As described above, T-*sukumo* and D-*sukumo* produced fast-and slow-changing microbial communities, respectively (Supplementary Figure S5). In order to understand the differences in the microbiota and metabolic functions, microbial community analysis and metagenome prediction were performed (data not shown). Although there was not so much difference in the taxonomic diversity between T-*sukumo* and D-*sukumo*, the constituted microbial communities were quite different. Major constituents in the microbiota in D-*sukumo* were *Marinimicrobium alkaliphilum* (similarity:95.5%–95.7%; 16.8%) followed by *Luteimonas dalianensis* (97.8%–99.8%; 7.5%), unidentified class *Clostridia* (5.0%) and *Azoarcus pumilus* (94.0%–98.0%; 3.8%), while those of T-*sukumo* were uncultured order *Longimicrobium* (13.7%) followed by *Oceanobacillus luteolus*-like species (97.2%; 12.9%), and *Sinibacillus* *soli* (97.4%–97.7%, 9.1%). The differences in microbiota between the two *sukumo* may be attributed to the procedures and environments of production. For example, original material of D-*sukumo* contains stalk of knotweed, whereas T-*sukumo* uses only leaves of knotweed. T-*sukumo* was produced on an in-house earthen floor, which explains why it contained a high ratio of *Bacillaceae*. The low ratio of *Bacillaceae* in D-*sukumo* suggests that it was not produced on an earthen floor. Considering the metabolic functional differences, the subpathway of “cell growth” in T-*sukumo* was 10.7-fold higher than that in D-*sukumo*. The contribution of *Bacillaceae* to “cell growth” was very high (more than 95% to the total constituents).

## Discussion

In this study, we clarified the effect of *I. tinctoria* L. leaf powder (LP) on the microbiota, the changes in functional subpathways accompanying the acceleration of indigo reduction and the prerequisites for indigo reduction in the early stage of fermentation. To attain the objective, we changed the preparation in batches by adding LP or not (for the control) at the start of fermentation, and using two kinds of *sukumo* that were known to produce different time lags from the preparation of the fermentation to the initiation of indigo reduction under minimum fluctuation fermentation pH. LP was effective in promoting the initiation of indigo reduction in both T-*sukumo* and D-*sukumo*. The presented results suggest that the addition of LP promotes rapid changes to the anaerobic metabolism-based microbial flora. According to the changes in the predicted functions of the microbiota, the effect of LP intensified from day 1 to day 2 in both of the *sukumo* using batches in the early stage of fermentation. The initial effect of LP in the T-*sukumo*, which produced a rapid change in the microbiota, was more intense than that in the D-*sukumo*, which produced a slow change in the microbiota and the primary duration was until day 3, while the duration in the T-*sukumo* was until day 5. Deduction from cellular metabolisms based on the constituted microorganisms suggests that stimulatory effects on the microbiota (i.e., oxygen-removing effect) by LP and consequent environmental pressures (i.e., decrease in ORP and the stimulatory effect of phytochemicals) brought rapid microbiota changes favorable to indigo reduction. The reduction in indigo may be initiated through the drastic changes in microbiota and the presence of appropriate amount of substrates that derived from *sukumo* and transitional change of the microbiota. It assumed that the indigo reduction occurs when the carbohydrate metabolism pathway for the extracellular electron transfer exceeds the threshold of the metabolic capacity in the microbial community.

To fulfill the prerequisite, a rapid substitution oxygen-metabolizing *Bacillaceae* by non-oxygen-metabolizing *Bacillaceae* (e.g., *A. macyae*-like bacteria), which greatly contribute to carbohydrate metabolisms is important and LP assists such a desirable change in the microbiota. A previous study reported that a stable state under alkaline anaerobic conditions, that induced dominance of the obligate anaerobe *Proteinivoraceae* was an important factor for maintaining of indigo fermentation for long period (Tu et al., 2019b). However, even in anaerobic conditions dominated by family *Proteinivoraceae*, which is equivalent to genus *Alkalicella*, at the beginning of the fermentation, indigo reduction was not initiated (Lopes et al., 2021a). In the present study, we confirm the more specific prerequisite phase transition of microbiota that is required for indigo reduction. The accompanying metabolic changes in the substrates, especially carbohydrates, and the contribution of genus *Alkalicella* to the carbohydrate metabolisms, were not high. Therefore, it is quite possible that it will be difficult to exceed the metabolic threshold for activating extracellular electron transfer even in the high ratio of genus *Alkalicella* in the microbiota.

The results of analysis of microbiota using T-*sukumo* indicated that *A. macyae*-like bacteria contributed to indigo reduction. In a previous study, *Alkalihalobacillus alkalinitrilicus* (reported as a strictly aerobic bacterium; Sorokin et al., 2008) was considered to contribute to indigo reduction (Lopes et al., 2021a). These strains, belonging to *Alkalihalobacillus* spp., are capable of adapting to vary low ORP conditions (lower than-600 mV). In addition, we observed another low ORP-adapted *Alkalihalobacillus* spp., which is reported to be an indigo reducing strings in mainly LP-added D-*sukumo* batches (Figure 3). Since these are facultative anaerobes, our results indicated that some of the facultative anaerobic *Alkalihalobacillus* spp. can adapt very low ORP, and have high possibility to reduce indigo. Although we have not investigated the indigo reducing *Alkalihalobacillus* spp. in detail, the strains may play an important role in indigo reduction in both the late fermentation phase as well as the early fermentation phase. Figure 1 shows indigo-reducing species in blue letters. Although *S. cohnii* was the dominant taxon in both batches T1 and T2 at the beginning of fermentation, they hardly contributed to indigo reduction. As a result of metagenomic prediction, the species showed a high contribution to abundance ratio in the metabolism of substrates, etc., than the obligate anaerobic microorganisms. This finding possibly indicated that the species did not fulfill the functions that are necessary to initiate an indigo reduction in the indigo fermentation fluid environment (i.e., the available substrates and physicochemical conditions). On the other hand, deeper dyeing can be seen only after the formation of a microbiota that is suitable for reducing indigo. This probably meant that establishment and maintenance of the indigo reduction requires several physiological functions.

The effect of LP on the early phase of D-*sukumo* fermentation and the later phase of T-*sukumo* fermentation was demonstrated in the previous study (Lopes et al., 2021a). In the previous study, although the initiation of indigo reduction was accelerated in the fermentation batch with added LP, the dyeing intensity tentatively decreased at day 25. This was probably due to the exhaustion of readily utilizable substrates in *sukumo*. Later, bacteria which are able to utilize cellulose or xylan appeared and the dyeing intensity recovered. Thus, it is thought that presence of available substrates, which are the sources for production of the reducing power for indigo, will be necessary to maintain the indigo reducing state. Therefore, we established the batch to which wheat bran was added at day 4 with the aim to maintain the presence of the substrates and the induction of bacteria, which are able to utilize cellulose or xylan (hard-to-utilize substrates). In the present study, relationships between *sukumo* materials and the additives of LP and wheat bran were demonstrated under minimum fluctuation of fermentation pH. There is a possibility that the substrates derived from *sukumo* were wasted under the moderate high pH. The reduction in ORP was much faster in T-*sukumo* than in D-*sukumo*. Normally, the ORP in indigo fermentation is reduced by oxygen metabolism by the facultative anaerobes derived from the *sukumo*. However, there was a large difference in the microbial community between D-*sukumo* itself and the initially appearing microbiota at day 1 in the fermentation. Therefore, readily utilizable substrates involved in *sukumo* may be consumed more when using D-*sukumo* than T-*sukumo*. In addition, a slow decrease in ORP allows microorganisms to consume of readily utilizable substrates derived from *sukumo* by oxygen metabolizable bacteria to an even larger extent. It is considered that the faster initiation of the indigo reduction may suppress the loss of the intrinsic substrates originally present in *sukumo*.

In addition, continuous moderate high pH (less than pH 11) maintenance may allow to increase unfavorable bacteria for indigo reduction by the microbiota. Therefore, although the microbiota possibly exhibited dyeing at day 5 in the D2 batch, it exhibited only faint dyeing. Further, addition of wheat bran led to faint dyeing in batches D4 and D3, within one or 2 days, respectively, after the addition. Rapid consumption of readily utilizable substrates at the beginning of fermentation may not have occurred in batches based on T-*sukumo*. Alternatively, there is a possibility that T-*sukumo* originally contained more readily utilizable substrates than D-*sukumo*. Therefore, rapid ORP reduction by oxygen metabolizing bacteria occurred in the T1 batch in the absence of LP. Microbial analyses of D-*sukumo* fermentation showed the appearance of a high ratio of *S. cohnii* followed by its rapid decrease then succession by obligate anaerobes, which may have prevented rapid consumption of the readily utilizable substrates. The large microbial flora change may have occurred because the LP addition accelerated the transition of microbial flora.

Concerning the successful example of addition of LP to T-*sukumo*, the effect of LP could be explained by the sharp drop in ORP. Notwithstanding the sharp decrease in *S. cohnii*, *Bacillus taeanensis*-like bacteria (98.4%–98.6%) remained in the fermentation batches that used D-*sukumo*. This was probably due to the high tolerance of *B. taeanensis*-like bacteria to the stress induced by the decrease in ORP and by the LP itself. However, the bacteria markedly decreased following the addition of wheat bran. This effect on indigo reduction suggests that owing to lowering of waste of readily utilizable substrates in *sukumo*, release of substrates and electron carriers from the deceased cells, and correction of bacterial metabolisms toward carbohydrates as the central substrates. The importance of effective use of the substrates for the reduction in indigo may be related to the observation in previous studies that indigo reduction decreased as the microbial diversity increased (Tu et al., 2019b, 2021). Although it is generally accepted that microbial diversity brings stability to the microbial community (Shi et al., 2022), unnecessary increase in the diversity of the microbial community in indigo fermentation will lead to substrate loss for the direction of the extracellular electron transfer.

In indigo fermentation based on D-*sukumo*, several phase transitions occurred in the course of initiation of reduction. Adding LP made the first phase transition more intensive. Although there was no hard evidence for cell disruption among the bacterial cells in the indigo fermentation, the decrease in the bacterial presenting ratio suggested that disruption of cells was triggered by the external environmental change. There is a possibility that LP plays a role of enhancing the trigger for cell disruption by adding additional stress to the bacterial cells, because the phase transition of the microbiota in the T-*sukumo* experiment cannot be explained by the ORP lowering effect alone. Indeed, it has been reported that *Indigofera tinctoria* L. contains polyphenol, tannins, and flavonoids (Sharma et al., 2016; Srinivasan et al., 2016). These constituents exhibit antimicrobial activities (Cushnie and Lamb, 2005; Turkmen et al., 2007; Ukoha et al., 2011). Probiotic effects of polyphenol and flavonoids have also been reported previously (Chen et al., 2019; Kumar Singh et al., 2019; Pei et al., 2020).

As a consequence of phase transition of the microbiota caused by rapid ORP change and antimicrobial phytochemicals, the oxygen metabolizable bacteria let decease in ratio. If some chemicals involve triggering to induce explosive cell lysis, the event may be affected in the later phase of the fermentation. Explosive cell lysis is mediated through the activity of cryptic prophage endolysin produce membrane vesicles derived from bacterial membrane, extracellular DNA, cytosolic proteins, and quorum sensing molecules (Turnbull et al., 2016; Toyofuku et al., 2017). In addition, electron mediators such as flavine are released from deceased cells. The released electron mediators accelerate the extracellular electron transfer mechanisms reported in Gram positive bacteria (Light et al., 2018, 2019). Thus, studies of the microbial community of indigo fermentation will provide a clue to the meaning of transitional changes and the maintenance of the ecosystem.

## Conclusion

Selection of necessary microorganisms which contribute indigo reducing metabolisms in the microorganisms involving in *sukumo* is indispensable for initiation of indigo reduction. This selection will lead to a correction of the metabolic pathways from utilization of substrates to production of extracellular reducing forces for the indigo reduction. For efficient use of limited substrates in the fermentation batch (primarily from *sukumo*), loss of available substrates due to consumption by microorganisms that do not contribute to indigo reduction should be limited. The addition of LP was effective for promoting the transitional changes to microbial community that contribute for anaerobic metabolisms and selection of the bacterial species which contribute indigo reduction. In employment of D-*sukumo*, in which rapid initiation of indigo reduction is difficult, the addition of wheat bran (in addition to the introduction of LP) was effective for accelerating the initiation of reduction by induction. The mechanisms were by inducing further positive changes in traditional microorganisms for indigo reduction, and introducing wheat bran as a substrate for the microorganisms which are able to reduce indigo. In addition, although in general, regulation of microbial communities toward favorable direction is thought to be difficult and there are not many such cases reported; in this study we demonstrated the effects of LP and wheat bran in deferent states of microbiota to promote the microbiota toward a favorable direction.

## Data availability statement

The datasets presented in this study can be found in online repositories. The names of the repository/repositories and accession number(s) can be found at: https://www.ddbj.nig.ac.jp/, DRA014240.

## Author contributions

HL, HS, and IY conceived and designed the experiments. ZT and HL performed the experiments. HL, ZT, and IY analyzed the data. IY wrote the manuscript. All authors contributed to the article and approved the submitted version.

## Funding

This work was supported by the Institute for Fermentation (IFO), Osaka (G-2020-3-035; to IY).

## Conflict of interest

The authors declare that the research was conducted in the absence of any commercial or financial relationships that could be construed as a potential conflict of interest.

## Publisher’s note

All claims expressed in this article are solely those of the authors and do not necessarily represent those of their affiliated organizations, or those of the publisher, the editors and the reviewers. Any product that may be evaluated in this article, or claim that may be made by its manufacturer, is not guaranteed or endorsed by the publisher.
