# Supplementary material for: Indigofera tinctoria L. leaf powder promotes initiation of indigo reduction by inducing of rapid transition of the microbial community
Source: Front Microbiol. 2022 Aug 9;13:957809. doi: 10.3389/fmicb.2022.957809 (PMC9395713; doi:10.3389/fmicb.2022.957809)
Supplement: Supplementary file 1 [file Table_1.docx]

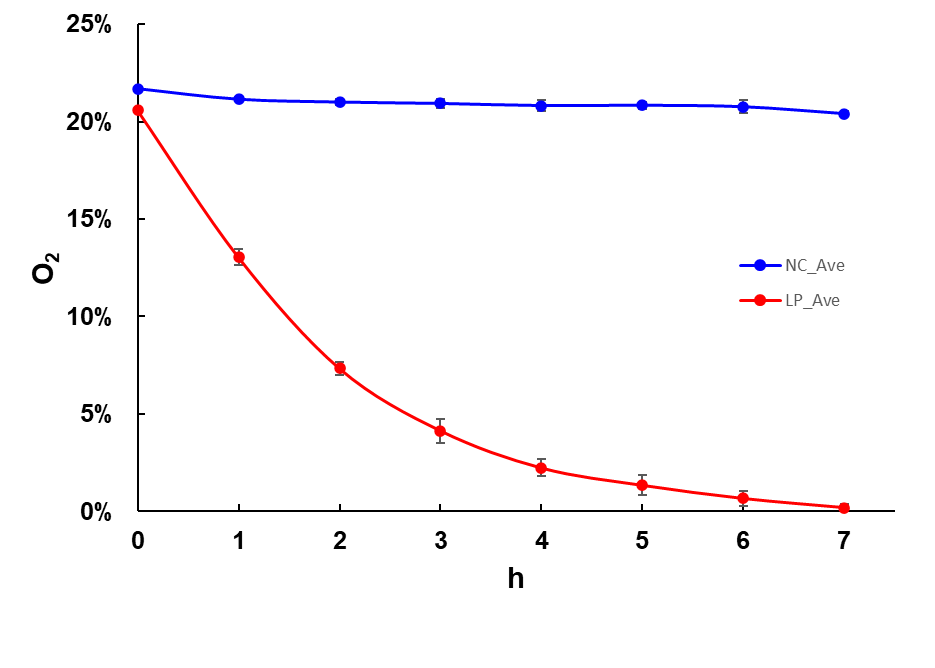


**Figure S1.** Oxygen consumption profile in wood ash extract by the effect of *Indigofera tinctoria* L. leaf powder (LP). LP (2 g) was added to 40 mL of wood ash extract in the sealed tube. Red: LP added; blue: non-LP added Control.


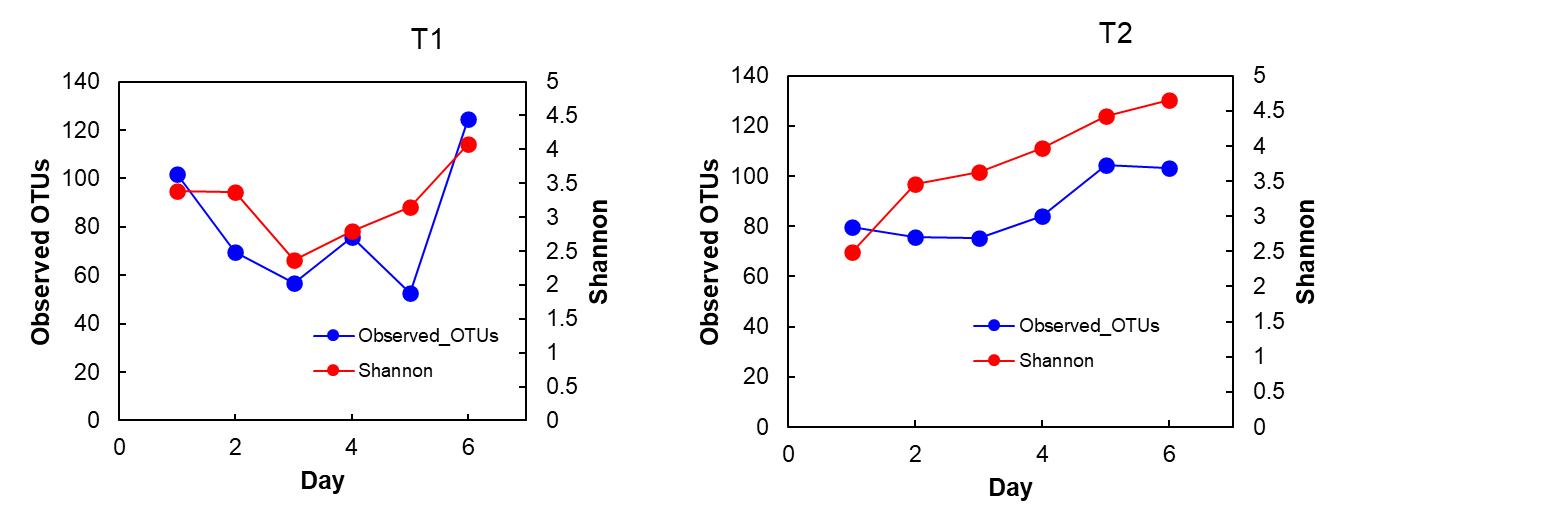


(B)

(A)

**Figure S2.** Changes in alpha diversity (blue circles: observed operational taxonomic units [OTUs]; red circles: Shannon index of diversity) depending on the fermentation period, analyzed using the Divisive Amplicon Denoising Algorithm (DADA2) based on the duration of period in indigo fermentation using T-*sukumo* in *Indigofera* *tinctoria* L. leaf powder (LP)-added samples (T2; [B]) and in the Control (T1; [A]). The 16S rRNA sequencing depth was 10,886 for each sample. The number corresponding to depth was according to the read number for the coverage of all samples. The values are average of n=10 and the standard deviations are less than 2.05 in observed OTUs and 0.028 Shannon index.


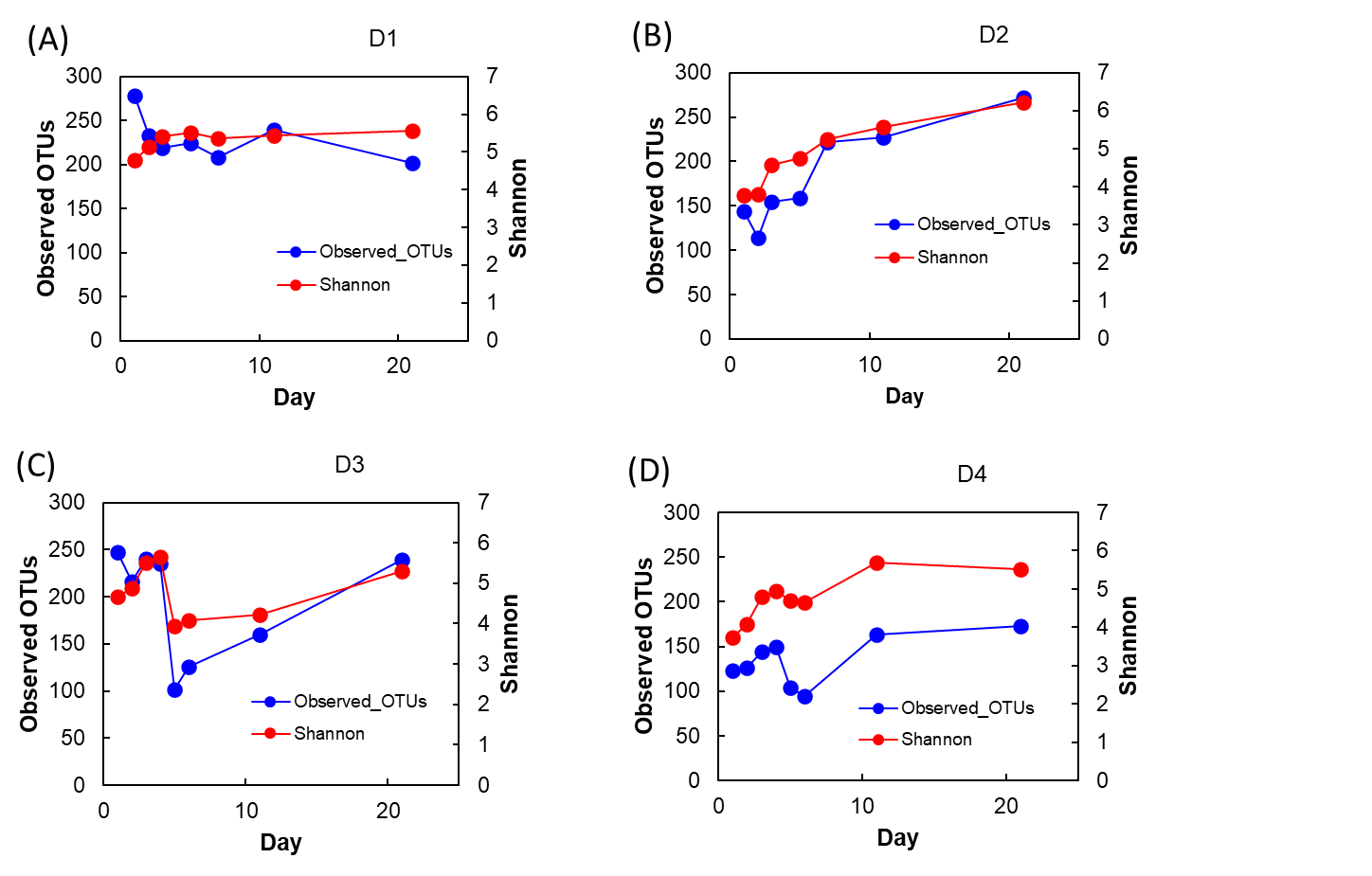


**Figure S3.** Changes in alpha diversity (blue circles: observed operational taxonomic units [OTUs]; red circles: Shannon index of diversity) depending on the fermentation period, analyzed using Divisive Amplicon Denoising Algorithm (DADA2) based on the duration of indigo fermentation using D-*sukumo* with *Indigofera tinctoria* L. leaf powder (LP) added (D2: [B]; D4 [D]) and Control (D1: [A]; D3 [C]). Wheat bran was added to batches D3 and D4 at day 4. The 16S rRNA sequencing depth was 12,851 for each sample. The number corresponding to depth was according to the read number for coverage of all samples. The values are average of n=10 and standard deviations are less than 2.70 in observed OTUs and 0.024 Shannon index.

**Figure S4.** The changes in the abundance of functional subpathways enhanced at day 21 on batch D4. Batch D4 (*Indigofera tinctoria* L. leaf powder [LP] added) used D-*sukumo*. Wheat bran was added in batch D3 and D4 at day 4. The metagenomic predictions were produced by PICRUSt2 and BURRITO. The abundance change expressed within each subpathway by high and low are denoted as red and blue, respectively.

**Figure S5.** Relative abundance of bacterial communities (≥1.0% in any sample) in T-*sukumo* and D-*sukumo* based on 16S rRNA analysis. The percentages in the brackets indicate the similarities with the known species in the NCBI database. Classification hierarchy depends on database similarity as follows: O, order level (<90% similarity); F, family level (≥90, <95%); G, genus level (≥95, <98.6%); and S, species level (≥98.6).
